# Supplementary material for: PhytoAFP: In Silico Approaches for Designing Plant-Derived Antifungal Peptides
Source: Antibiotics (Basel). 2021 Jul 5;10(7):815. doi: 10.3390/antibiotics10070815 (PMC8300835; doi:10.3390/antibiotics10070815)
Supplement: Supplementary file 1 [file antibiotics-10-00815-s001.zip › antibiotics-1241099-supplementary.pdf]

Additional Information

Competing financial interests

The authors declare no competing financial interests.

Figure Legends

Figure S1. NC-5 termini (split amino acid composition) of PhytoAFP and Non-PhytoAFP.

Figure S2. NC-10 termini (split amino acid composition) of PhytoAFP and Non-PhytoAFP

Figure S3. CN-5 termini (split amino acid composition) of PhytoAFP and Non-PhytoAFP

Figure S4. CN-10 termini (split amino acid composition) of PhytoAFP and Non-PhytoAFP

Figure S5. Overall physicochemical composition of PhytoAFP and Non-PhytoAFP

Figure S6. NC-5 termini (physicochemical composition) of PhytoAFP and Non-PhytoAFP

Figure S7. NC-10 termini (physicochemical composition) of PhytoAFP and Non-PhytoAFP

Figure S8. CN-5 termini (physic-chemical composition) of PhytoAFP and Non-PhytoAFP

Figure S9. CN-10 termini (physicochemical composition) of PhytoAFP and Non-PhytoAFP

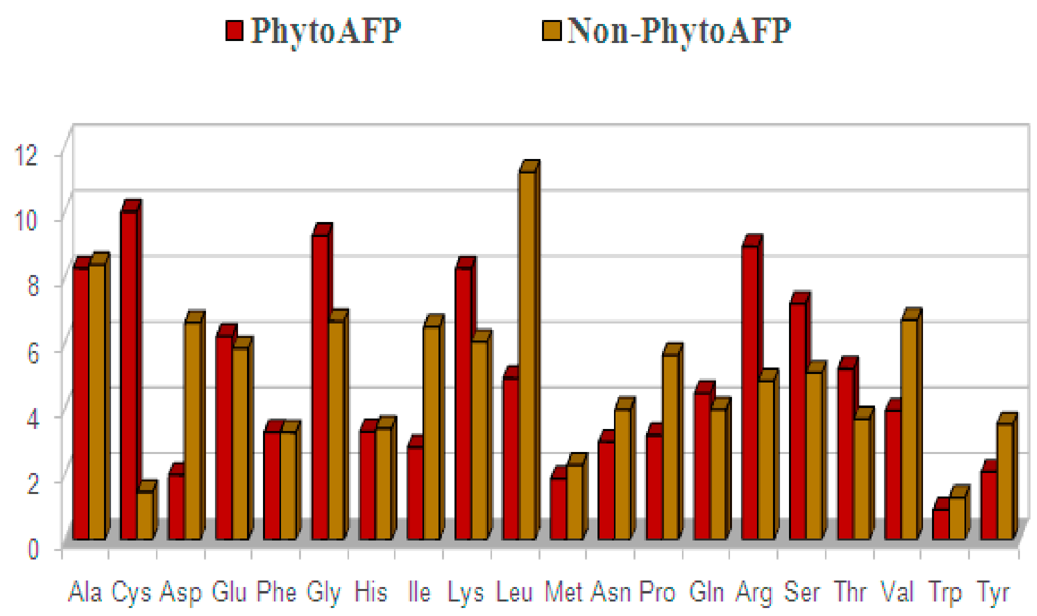

Figure S1. NC-5 termini (split amino acid composition) of PhytoAFP and Non-PhytoAFP

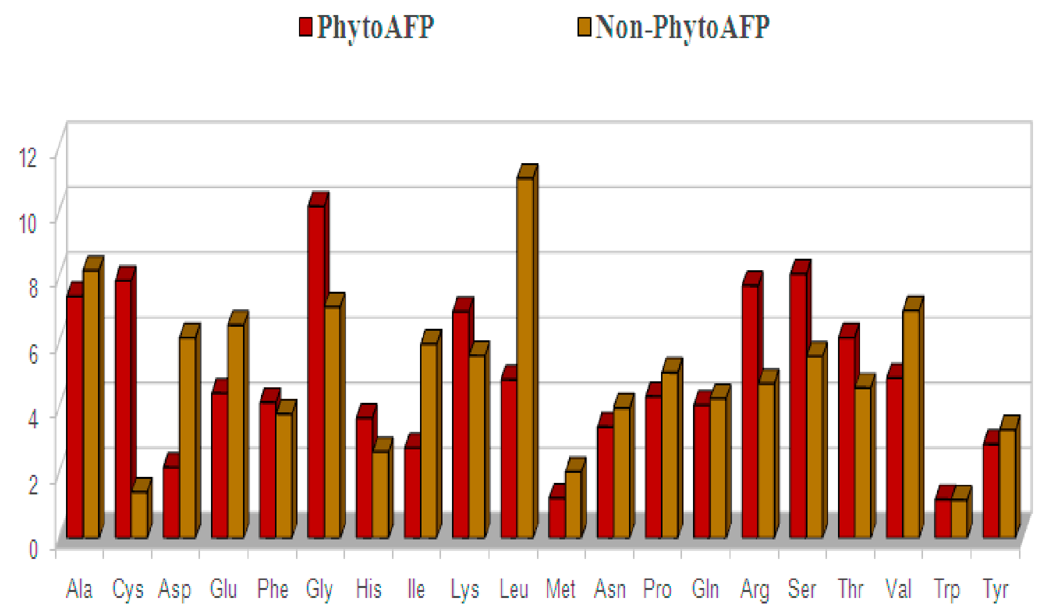

Figure S2. NC-10 termini (split amino acid composition) of PhytoAFP and Non-PhytoAFP

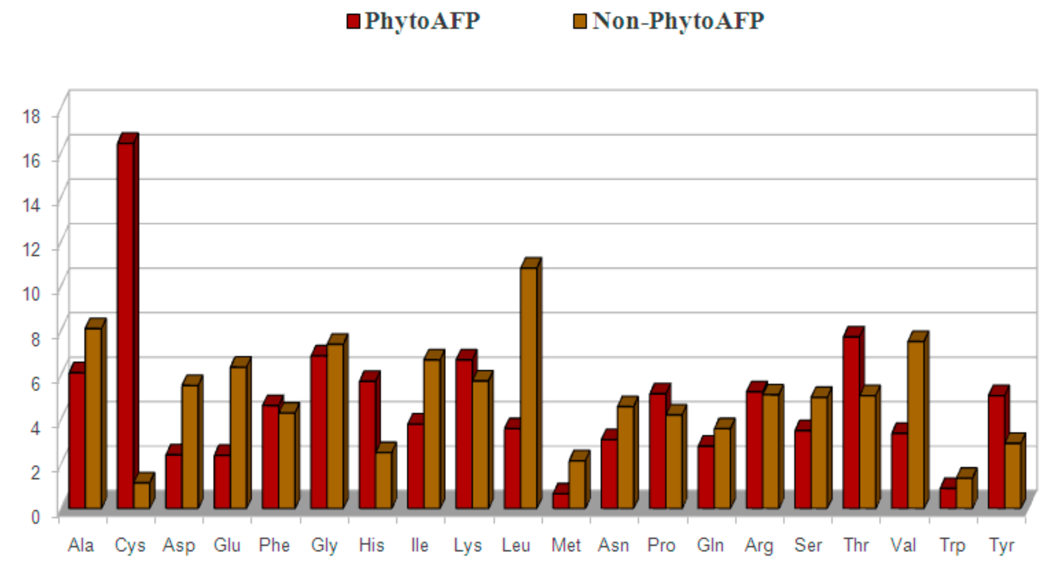

Figure S3. CN-5 termini (split amino acid composition) of PhytoAFP and Non-PhytoAFP

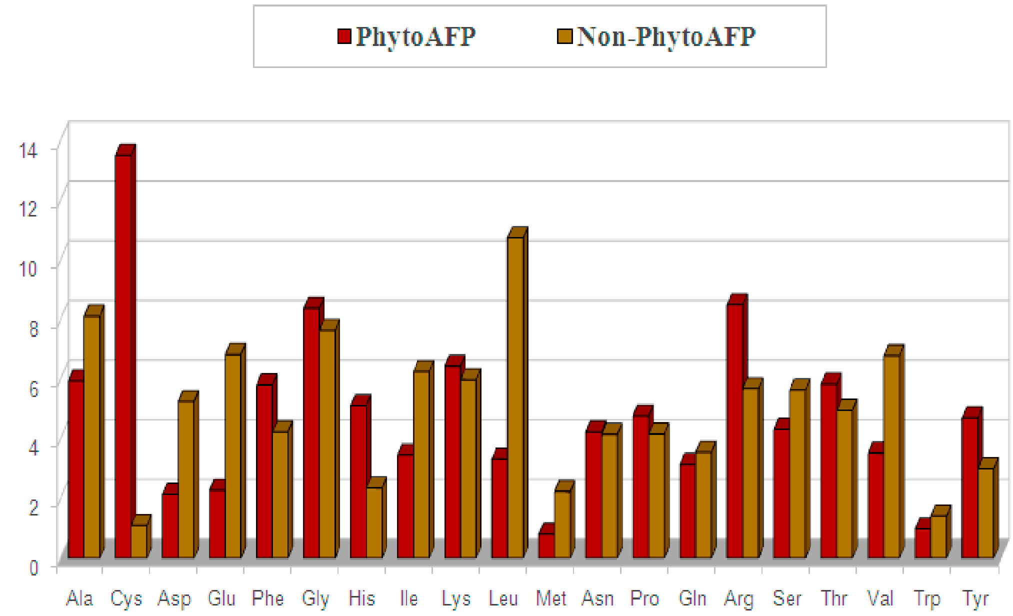

Figure S4. CN-10 termini (split amino acid composition) of PhytoAFP and Non-PhytoAFP

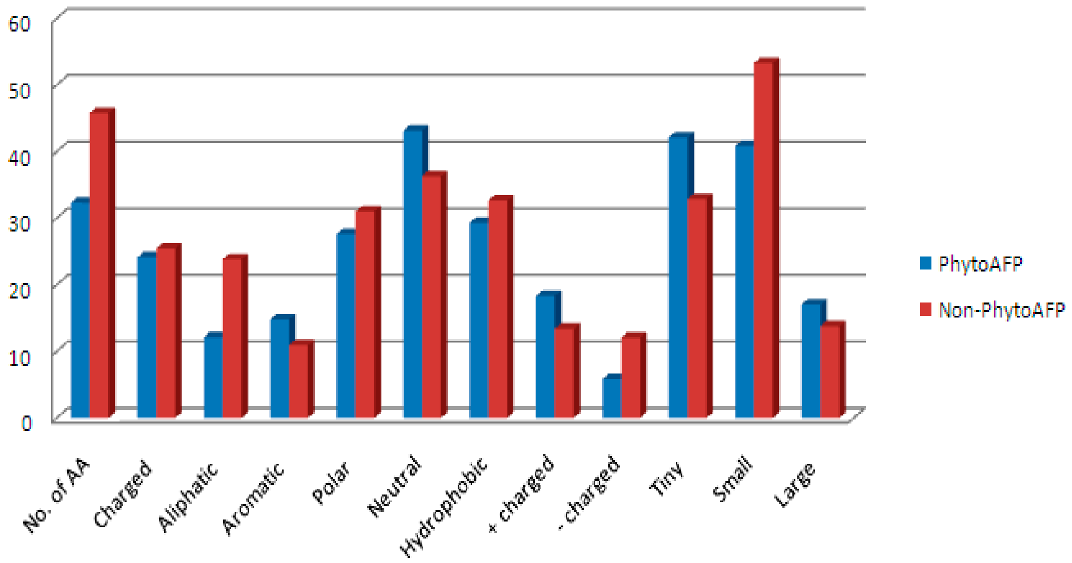

Figure S5. Overall physicochemical composition of PhytoAFP and Non-PhytoAFP

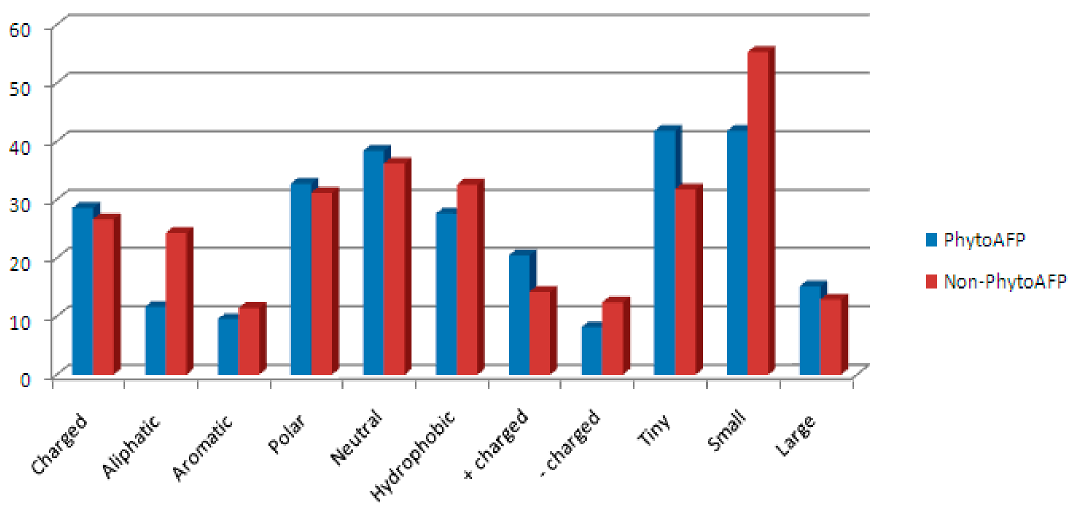

Figure S6. NC-5 termini (physicochemical composition) of PhytoAFP and Non-PhytoAFP

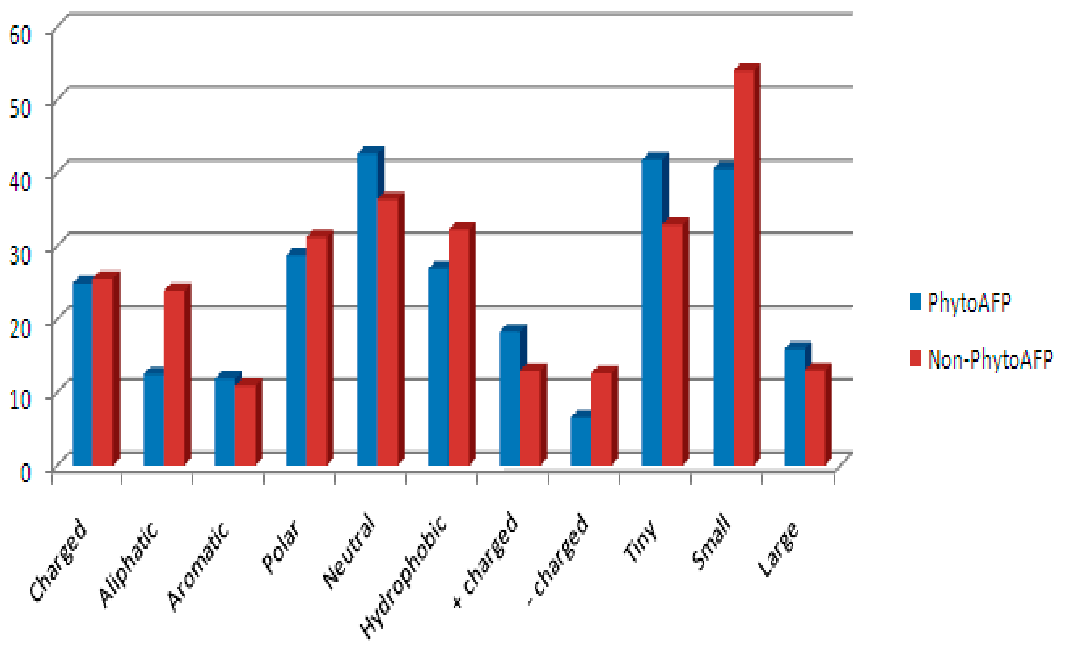

Figure S7. NC-10 termini (physicochemical composition) of PhytoAFP and Non-PhytoAFP

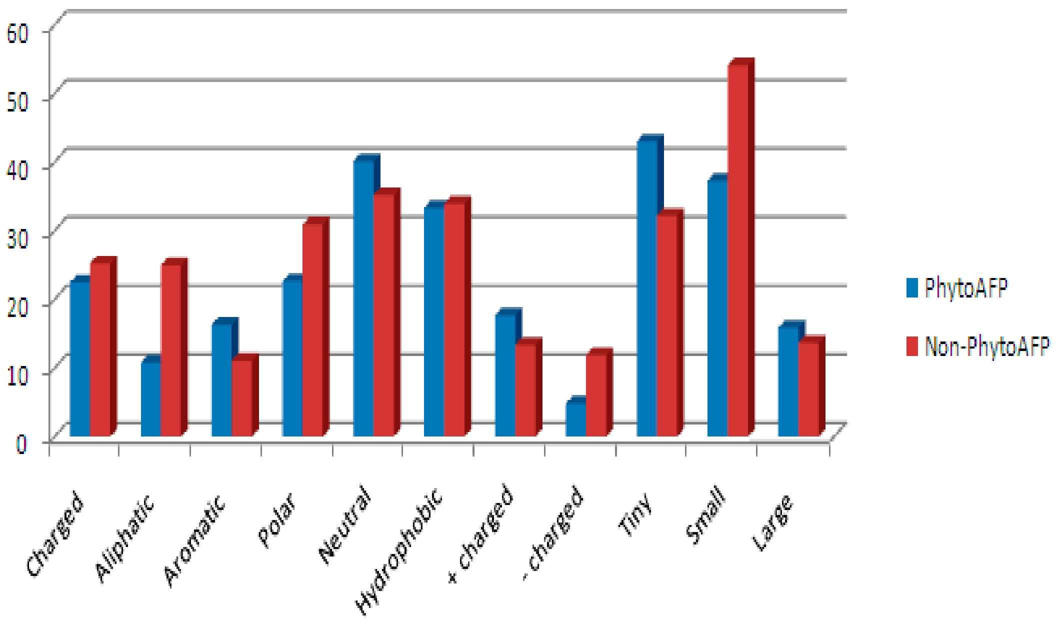

Figure S8. CN-5 termini (physic-chemical composition) of PhytoAFP and Non-PhytoAFP

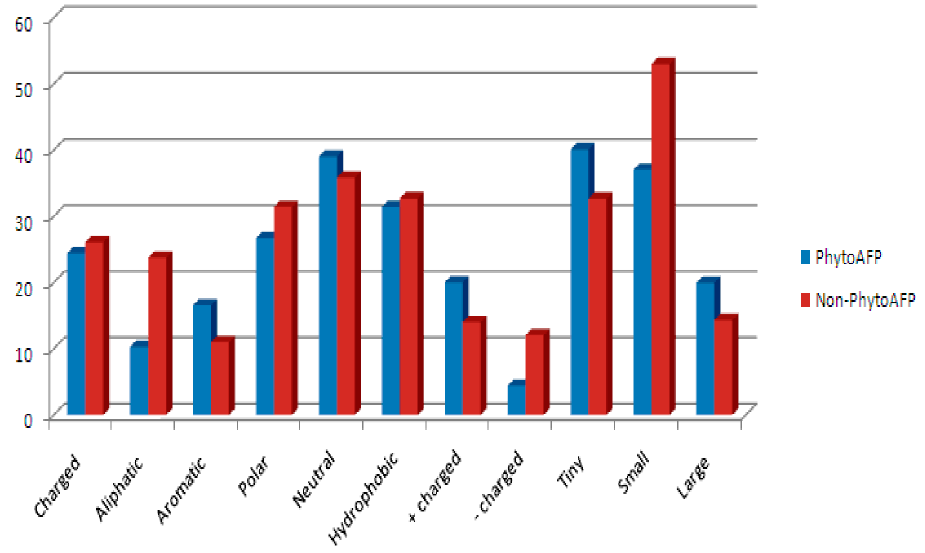

Figure S9. CN-10 termini (physicochemical composition) of PhytoAFP and Non-PhytoAFP

Table S1. Exclusive motifs of PhytoAFP and Non-PhytoAFP dataset

Sr. No.	Positive motifs	Negative motifs
1	NYVF	LT
2	NYVFP	TL
3	YVFP	TG

4	NYVFPA	AT
5	VFPA	TA
6	YVFPA	VT
7	FPAH	TV
8	NYVFPAH	GT
9	VFPAH	TE
10	YVFPAH	ST
11	PAHK	KT
12	FPAHK	IT
13	ARHG	TI
14	ARHGS	TS
15	GSNY	ET
16	NYVFPAHK	TP
17	SNYV	TR
18	VFPAHK	DT
19	YVFPAHK	FT
20	CFC	TT
21	SNYVF	TD
22	GSNYV	TK
23	GSNYVF	PT
24	SNYVFP	TF
25	HGSN	QT
26	HGSNY	TQ
27	RRC	RT
28	HGSNYV	NT
29	GSNYVFP	YT
30	HGSNYVF	TY
31	SNYVFPA	HT
32	KARHG	TN
33	KARHGS	TH
34	RHGSN	WL
35	RHGSNY	LAL
36	GSNYVFPA	KM
37	HGSNYVFP	TM
38	RHGSNYV	VLE
39	RCF	ALK
40	RCFC	TW
41	RHGSNYVF	AAE
42	SNYVFPAH	LAT
43	CQE	LLD
44	HGSNYVFPA	LLE
45	ARHGSN	LTE
46	ARHGSNY	TC
47	CASV	VIA
48	GSNYVFPAH	VLA
49	NCAS	WN
50	RHGSNYVFP	
